# Role of Claudin Proteins in Regulating Cancer Stem Cells and Chemoresistance-Potential Implication in Disease Prognosis and Therapy

**DOI:** 10.3390/ijms21010053

**Published:** 2019-12-20

**Authors:** Saiprasad Gowrikumar, Amar B. Singh, Punita Dhawan

**Affiliations:** 1 Department of Biochemistry and Molecular Biology, University of Nebraska Medical Center, Omaha, NE 68198-5870, USA; sai.gowrikumar@unmc.edu (S.G.); amar.singh@unmc.edu (A.B.S.); 2 VA Nebraska-Western Iowa Health Care System, Omaha, NE 68105, USA; 3 Department of Biochemistry and Molcular Biology, Fred and Pamela Buffet Cancer Center, University of Nebraska Medical Center, Omaha, NE 68105, USA

**Keywords:** claudins, cancer, stem cell, chemoresistance

## Abstract

Claudins are cell–cell adhesion proteins, which are expressed in tight junctions (TJs), the most common apical cell-cell adhesion. Claudin proteins help to regulate defense and barrier functions, as well as differentiation and polarity in epithelial and endothelial cells. A series of studies have now reported dysregulation of claudin proteins in cancers. However, the precise mechanisms are still not well understood. Nonetheless, studies have clearly demonstrated a causal role of multiple claudins in the regulation of epithelial to mesenchymal transition (EMT), a key feature in the acquisition of a cancer stem cell phenotype in cancer cells. In addition, claudin proteins are known to modulate therapy resistance in cancer cells, a feature associated with cancer stem cells. In this review, we have focused primarily on highlighting the causal link between claudins, cancer stem cells, and therapy resistance. We have also contemplated the significance of claudins as novel targets in improving the efficacy of cancer therapy. Overall, this review provides a much-needed understanding of the emerging role of claudin proteins in cancer malignancy and therapeutic management.

## 1. Introduction

### 1.1. Tight Junctions

Tight junctions (TJs) are the sites where tissues interface directly with the external environment or internal compartments that are contiguous with the external environment and are lined by mucosal surfaces, where epithelial cells act insulation for the internal organ. These structures not only provide a protective layer but also act as a selective barrier between the body and the gut lumen that restricts free exchange across the paracellular space [1,2]. There are three main transport mechanisms across the epithelial layers, which include the trans-cellular pathway (passive diffusion), carrier dependent pathway (carrier or receptors), and the paracellular pathway (passage through spaces between cells). Among these transport mechanisms, the apical junctional complex, a crucial factor for the paracellular pathway, is composed of three junctions from apical to basal are known as the tight junction (zonula occludens), adherens junction (zonula adherens), and desmosome (macula adherens) [3]. The TJs are intercellular junctions, which act as permeability barriers in epithelial cells [1]. The tight junction proteins are diverse and include occludins (the first one to be found), claudins, tricellulin, cingulin, and junctional adhesion molecules (JAM). These proteins interact within themselves and with the cellular cytoskeleton to form a complex architecture [4,5,6,7,8]. Among these TJ proteins, claudins are key proteins, acting as both pores and barriers, aiding the paracellular pathway between epithelial cells [9,10].

### 1.2. Claudins

The functionalities of claudins are as follows: (1) Fence function, responsible for maintaining polarity by differentiating apical and basolateral cell domains; (2) Signaling molecule, involved in cell growth, survival, proliferation, and differentiation; (3) Barrier function, this gate function separates compartments with fluids to avoid intermixing [11]. Claudins were identified as a major integral membrane protein by Tsukita and his colleagues in 1998, before which the only known tight junction protein was occludin [12,13]. Studies conducted to overexpress claudins in fibroblasts, which do not have tight junctions, were able to reconstitute tight junction-like networks of strands, which shows the importance of claudins in tight junction assembly [14]. Several claudin isoforms have been identified in mammals. These have high sequence homology in the first and fourth transmembrane domains and extracellular loops. Further, the homologous classic claudins include claudins 1–10, 14, 15, 17, and 19, and non-classical claudins comprised of claudins 11–13, 16, 18, and 20–27, which are less homologous [15]. 

**Figure 1:**
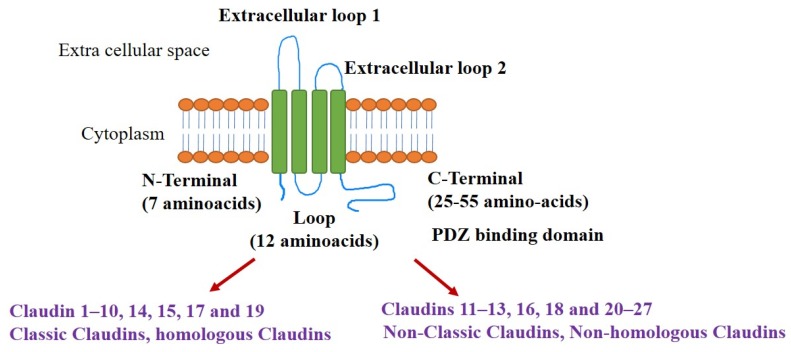
Structural organization of claudin proteins (monomer), and its classification based on homologous sequences between them. Colour code: Green- transmembrane domains; Orange: Bilipid layer, Blue–Extracellular loops/N and C termini.

The structure of claudins is comprised of four transmembrane domains, the intracellular N and C termini, and the two extracellular loops (ECLs). The claudin structure encompasses N-termini (7 amino acids), C-termini (25–55 amino acids), and loops containing 25–55 aminoacids. The ECLs are involved in barrier and pore formations. There are two ECLs, ECL1 consists of ~50 amino acids with two conserved cysteines involved in the barrier function. Negative and positive charges in ECL1 contribute to pore formation. The schematic representation of the structure of claudins and its classification is depicted (Figure 1). The ECL2 is responsible for homo and heterotypic interactions and was recently shown to be involved in host cell binding and cytotoxicity for the *Clostridium perfringens* enterotoxin. The ECL2 usually has ~25 amino acids, but fewer in claudin-11 and more in claudin-18 [16]. Claudins interact with other TJ-associated proteins through carboxy-terminal tails, which contain a PDZ-domain binding motif [17].

## 2. Claudins as Oncogenic Signal Transducer

The expression of claudins varies among different tissue types [18]. As an important structure in regulating paracellular permeability, claudin overexpression influences trans-epithelial resistance (TER) and ion permeability [19,20,21,22]. Aberrant expressions of claudins have been reported in various cancers. Some of the claudins known to be frequently dysregulated in cancers are claudin-1, -3, -4, and -7 [23]. A large body of evidence highlights claudins as pro and anti-tumorigenic factors [24,25,26,27,28,29,30,31]. The potential of claudins to act as proto-oncogene or tumor promotor in various cancers are summarized in Table 1. In addition, several recent studies have also demonstrated the importance of claudins as tumor suppressors [24,25,26,27,28,29,30,31]. A recent study by Chang et al. in 2019 provided evidence for intestinal hyperplasia and adenomas in claudin-7 knockdown mice [32]. Consistent with this, claudin-7 was downregulated in colon cancer patient samples as compared to normal tissue [33]. These effects of claudin-7 were achieved by inhibiting phosphorylation and nuclear localization of Akt. Conversely, claudin-7 association with Epithelial cell adhesion molecule (EPCAM) supports proliferation, upregulation of anti-apoptotic proteins, and drug resistance [33]. Claudin-18 knockout mice spontaneously developed lung adenocarcinomas, and its mRNA expression was decreased in lung adenocarcinomas. Claudin-18 inhibits Akt signaling through modulation of yes-associated protein/Taz (Yap/Taz) and insulin-like growth factor (IGF-1R) signaling in lung cancer [34]. Further, the depletion of claudin-3 induced tumor burden by enhancing β-catenin activity through (IL)-6/STAT3 signaling in colon cancer [35]. Yet another study by Che et al. in 2018 [36] identified claudin-3 as a suppressor of lung squamous cell carcinoma cells, in which overexpression of claudin-3 inhibited invasion, migration, and EMT of lung squamous cell carcinoma. Similarly, claudin-4 accelerates cell migration and invasion in ovarian tumor cell lines, in support of this, peptide-mediated silencing of claudin-4 in ovarian cancer cells exhibited lower tumor burden [37]. Claudin-6 was shown to be a tumor suppressor through genetic manipulation studies in cervical carcinoma cells wherein loss of claudin-6 exacerbated cell proliferation and tumor growth [38,39]. An array of articles from Dhawan et al., have proved a significant role of claudin-1 as a tumor promoter in colon cancer [40,41]. In one of their reports, increased claudin-1 expression was causally associated with metastasis [40]. In contrast to claudin-1, claudin-7 has an inverse role on EMT, wherein it causes mesenchymal to epithelial transformation (MET) in Rab25 dependent manner to combat colon cancer [42]. Similarly, claudin-2 is upregulated in colon cancer and is involved in cancer progression. Claudin-2 suppression in colon cancer cells has led to decreased cell proliferation through the modulation of EGF signaling [43]. Opposite colon cancer, claudin-1 is frequently down-regulated in invasive human breast cancer. Recently, mutations of claudin-1 have been reported in breast cancer, which has led to claudin-1 transcript variants shorter than classical claudin-1 transcript [44]. Taken together, it appears that the deregulated claudin composition in any given epithelial cells sheet may modify the signaling and associated changes in protein partnering to modulate oncogenesis.

To glimpse how claudins can achieve its pro or anti-tumorigenic effect, understanding the regulation of claudins in normal and cancer cells is essential. Recently it has been demonstrated that claudins are not a static and rigid seal of the paracellular space; rather, they are dynamically capable of responding to various biochemical and mechanical stimuli through reshaping and remodeling [50,51]. Epigenetic regulation of claudins has recently gained significant importance. The claudin-3 promotor is known to possess low DNA methylation and high histone H3 acetylation for its expression in ovarian cancer cells [52]. DNA hypomethylation of the claudin-4 promotor is an important factor for its high expressions in gastric cancer [53]. Downregulation of claudin 1 via DNA promoter methylation is reported in estrogen receptor-positive breast cancer [54]. Claudins are also regulated at the transcriptional level by different transcription factors. A study has reported novel post-transcriptional regulation of claudin-1 in colon cancer cells [55], the authors documented the role of histone deacetylase (HDAC)-dependent histone acetylation as a key post-transcriptional regulation over claudin-1 expression, as found through HDAC inhibitor studies. Studies demonstrate the interaction of Slug and Snail (transcriptional factors) with the E-box element in the claudin-1 promoter causes inhibition of claudin-1. Snail is known to act as a transcription factor causing repression of E-cadherin (E-CAD) and has a potential role in promoting tumorigenesis. Slug is also a pivotal transcription factor involved in cell migration during embryogenesis and in tumor cell invasion and migration [56]. Yet another transcriptional factor known to be associated with claudin-1 is Runt-related transcription factor 3 (RUNX3), which is a gastric tumor suppressor [47]. Caudal homeobox proteins (Cdx1 & Cdx2) and GATA binding protein 4, GATA4) are known activators of claudin-1 promoters in colon cancer [57]. Sp1 is a transcriptional factor known to regulate claudin-3 and claudin-4 promoter activity in ovarian cancer [52,58]. Apart from these transcriptional regulations, claudins are also known to be regulated by post-translational modifications involved in their protein localization, interaction with other proteins, and overall turnover [59,60]. The post-translational modification of claudins includes palmitoylation, O-glycosylation, and phosphorylation [61,62]. Phosphorylation is one of the key regulatory modifications for the regulation of intracellular localization and degradation of claudins.

Claudins are phosphorylated by many different enzymes like protein-kinase A/C, protein phosphatase 2A and mitogen-activated protein kinase (MAPK) [63,64]. The localization or dissociation of claudins to TJs is regulated by phosphorylation. For phosphorylated claudin-1, -5, and -16 are localized in the TJs while in contrast, phosphorylated claudin-3 and -4 dissociate from TJs [64,65,66]. Furthermore, the rho family of small dimeric G proteins mediated phosphorylation of claudin-5 at T207 was recently reported [67]. The phosphorylation of claudin-1 at different serine sites (192, 205, 206, and T191) regulates its assembly at tight junctions [68]. The cAMP-dependent protein kinase (PKA) is known to phosphorylate of claudin-3 at amino acid 192 at the C terminus. Claudin-4 is phosphorylated by atypical PKC (aPKC) at serine195 [65]. Another important posttranslational modification playing a key role in claudin regulation is palmitoylation. Emerging articles have demonstrated the importance of palmitoylation in claudin localization into tight junctions. In claudin-5 self-assembly, palmitoylation restricts specific protein-protein conformations, as reported by Rajagopal et al. [61]. Claudin-7 interacts directly with EpCAM along the basal membrane. Palmitoylation regulates the ability of claudin-7 to interact with integrins, recruiting EpCAM, and concomitantly associate with the actin cytoskeleton [69].

## 3. Claudins and Stem Cells

Stem cells are crucial for the development and homeostasis of many different tissues. Stem cells are also involved in cell replacement therapies in the case of cell damage or degeneration [70]. Pluripotency of stem cells is defined as self-renewing and differentiating potential into all three germ layers. Human pluripotent stem cells are very promising in regenerative medicine. The stem cell further differentiates into a wide variety of cells under the influence of diverse signaling molecules, growth factors, and transcription factors [71]. Recent research is focused on understanding the signals, which maintain pluripotency or differentiation potential. Various intrinsic and extrinsic factors are involved in stem cell maintenance, self-renewal, and differentiation [71]. On the other hand, stem cells are also an important factor for many tumors. Dysregulated pluripotent stem cells in tumors are more aggressive and have the potential to reform the whole tumor [72]. Thus, it becomes important to selectively remove undifferentiated human pluripotent stem cells (hPSCs) from differentiated cultures. For achieving this, selective pluripotent-specific cell surface markers are needed, which can separate undifferentiating from the differentiated cells. While screening for a highly specific marker protein specific for the undifferentiated hPSCs, Uri Ben-David et al. [73] found claudin-6 to be highly specific for undifferentiated hPSCs. The expression of claudin-6 was 90-fold higher in undifferentiated hPSCs than in differentiated cells. The proof for the involvement of claudins in epithelial differentiation from embryonic stem cells was first reported by Sugimoto et al. [74], where the potential of claudin-6 to trigger epithelial morphogenesis in mouse stem cells was reported. Also, claudin-6 regulated other tight-junction and microvillus molecules claudin-7, occludin, Zonula occludens (ZO-1α+), and ezrin/radixin/moesin-binding phosphoprotein50, which strongly proved the role of claudins in epithelial differentiation [74]. This was further supported by other studies, which also showed the expression of claudin-6 is an early marker in embryonic stem cells [75,76]. Differentiation of Human Embryonic Stem Cells to Hepatocyte-Like Cells resulted in a decrease in stem cell markers Oct3/4 and Nanog as expected. Along with stem cell markers, claudin-1 declined eventually, whereas claudin-4 increased and was highest at the end stage of differentiation [77].

A growing body of evidence focuses on cancer stem cells in cancer biology. The drawbacks of cancer treatment failures and drug resistance are proved to originate from cancer stem cells, which are a small subpopulation in tumors. Recently the factors regulating cancer stem cells have gained significant importance and opened new avenues for targeted therapies and thus decrease the chance of recurrence of the disease [78]. Cancer stem cells (CSCs) represent a small group of cells in typically heterogeneous tumors, which possess tumor-initiating and self-renewal properties, giving rise to non-tumorigenic progeny. CSCs are enriched after chemotherapy and lead to therapy failure and thus recurrence of cancer. The role of CSCs in tumor relapse, metastasis, and therapeutic refractoriness is well described [79]. The role of claudins in cancer stem cell (CSC) biology is gaining much attention. The WNT pathway is well known to provide the key signals for achieving this particular phenotype. It is also established that the *Wnt* signal transduction pathway is important in normal and malignant stem cells [80]. Recent articles have highlighted the link between claudin and the *Wnt*/β-catenin signaling pathway and the role of CSC in this cross-talk. Claudin-1 and claudin-2 transcription is regulated by WNT*t* signaling, and they are known to regulate the β-Catenin- T-cell factor/lymphoid enhancer-binding factor (TCF/LEF) signaling pathway to regulate CSC [81,82]. In contrast, other claudins negatively regulate WNTsignaling cascades, such as loss of claudin-3 inducing WNT/β-catenin activation, thus aiding in the promotion of colon cancer [35]. Darido et al. provided evidence for Tcf-4 and Sox-9 regulating the expression of claudin-7 [46]. In addition, studies by Prat et al. discovered a new claudin-low molecular subtype of breast cancer [83]. The key characteristics of this subtype are low expression of tight junction and junction adherens proteins (claudin-3, -4 and -7, and E-cadherin), and enriched in stem cell and EMT features. Patients having high-grade invasive ductal breast carcinoma in this subgroup had a poor prognosis, absence of luminal differentiation markers, enhanced EMT markers, expression of immune response genes, and most closely resembled mammary epithelial stem cells. This suggested that low claudin cells might emerge from more immature stem or progenitor cells and comprise cancer stem cells. Thus, identification of the low claudin subtype in breast cancer has shown the potential of claudins in regulating stem cells. In addition, claudin-3 is known to play an oncogenic role in non-small cell lung cancer (NSCLC). One of the major contributing factors for the role of claudin-3 is regulation of cancer stemness and chemoresistance in non-squamous NSCLC. The depletion of claudin-3 was able to combat the formation of spheres and tumor formation as well as increased sensitivity to cisplatin [84]. Further, claudin-3 inhibition by small-molecule inhibitors including withaferin A, estradiol and fulvestrant, suppressed cancer stemness and combated chemoresistance, giving strong evidence for the role of claudin-3 in inducing stemness. Another claudin playing an important role in stem cell regulation is claudin-18 in lung cancer [85], which has been reported to have a role in the aberrant proliferation of alveolar epithelial type II (AT2) cells, resulting in lung enlargement and parenchymal expansion by restrictions on stem/progenitor cell proliferation. Recently, claudin-2 was shown to be restricted in the stem/progenitor cell compartment of intestinal crypts. It enriches aldehyde dehydrogenase ALDH^High^ cancer stem-like cells in heterogeneous colorectal cancer cell populations through the regulation of Yes-associated protein (YAP) activity and miR-222-3p expression [86]. Overall, these studies give an overview of the potential role of claudins in stem cell biology. The role of claudins in the regulation of stem cells is summarized in Table 2. The claudin mediated enrichment of stem cells provides a new axis-of-evil for a preferential therapeutic target, which has potential clinical consequences.

## 4. Claudins in Chemoresistance

**Figure 2:**
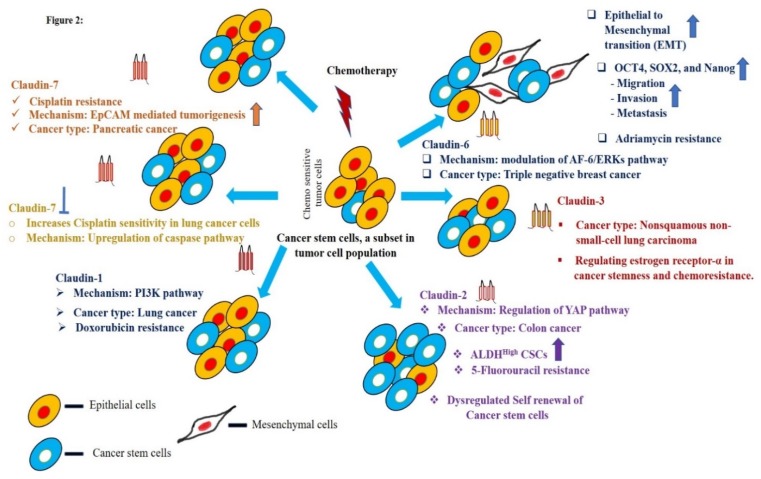
The central role of claudins in the regulation of epithelial to mesenchymal transition (EMT), cancer stem cells, and chemoresistance in various cancers. 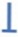
- inhibition of claudin-7. The arrows indicate the upregulation and higher enrichment of the mentioned signaling molecules, colour is respective of each claudin.

Most cancer patients initially respond to chemotherapy. Eventually, cancer relapses due to chemoresistance resulting in treatment failure causing death. The mechanisms of chemoresistance in cancers are still largely unknown [87]. Since the role of claudins in the regulation of cancer stem cells is well documented, their correlation with drug resistance and distant metastasis is inevitable and obvious [49,88,89]. In brief, claudin-3 and -6 are correlated with lymph node metastasis in squamous cell lung carcinomas [90,91]. Claudin-4 is highly expressed in primary and metastatic prostate cancer [92] and gastric cancer [93,94]. Claudin-1 and -7 have proved to have an inverse role in colon cancer, wherein claudin-1 elevates the metastasis of colon cancer cells. On the other hand, suppression of claudin-7 leads to liver metastasis [40,42]. Epithelial to mesenchymal transition (EMT) is a piece of vital machinery responsible for invasiveness and initiation of metastasis and chemoresistance of cancer cells. Claudins are known inducers of EMT in cancers. Claudin-1 is known to induce EMT in colon, liver, nasopharyngeal carcinoma, and breast cancers [40,95,96]. At the same time, claudin-7 is reported to be involved in establishing MET in colon cancer [36,42]. Claudin-3 suppresses EMT in lung cancer cells [36]. Overall, the potential role of claudins in EMT, Metastasis and CSC enrichment provides the rationale for exploring them as a key factor in establishing drug resistance. Claudins as chemo-resistance modulators is an emerging field of research. In a recent article, the potential of claudin-6 in enhancing chemoresistance to Adriamycin in triple-negative breast cancer (TNBC) was documented [97]. This effect of claudin-6 was mediated through its regulation over the AF-6/extracellular signal–regulated kinases (AF-6/ERK signaling pathway and up-regulation of cancer stem cells. Claudin-3 is also identified as a molecule to combat cisplatin chemoresistance in non-squamous lung carcinoma [84]. Here, claudin-3 overexpressing lung cancer cells were insensitive to cisplatin treatment compared to control cells. Adding to this, knockdown of claudin-3 or claudin-4 in ovarian cancer cells induced resistance to cisplatin by the regulation of Cu transporter CTR1 [98]. Another study by a Japanese group of researchers reported a high expression of claudin-4 in the ovarian cancer tissues of platinum-resistant patients [99]. In lung cancer, claudin-1 is a key deciding factor for metastasis and a responsible factor for drug resistance towards cisplatin through the up-regulation of Unc-51 Like Autophagy Activating Kinase 1 (ULK1) phosphorylation [100,101]. It is also known to enhance drug resistance in liver cancer cells by modulating autophagy to achieve drug resistance. The role of claudin-7 in drug resistance [102] has also been reported, wherein decreased drug resistance, increased apoptosis and diminished anti-apoptotic PI3K/Akt pathway was achieved by knocking down claudin-7, proving the potentiality in chemo-resistance [103]. It is well known that EpCAM associates with claudin-7 and is known to be involved in cancer metastasis. Florian et al. [69] have provided evidence for the increased migratory potential of pancreatic cancer cells upon EPCAM and claudin-7 association influencing cell-cell adhesion. Interestingly, the EPCAM and claudin-7 association seems to enhance drug resistance against cisplatin through enhancing MAPK and c-Jun N-terminal kinases (JNK) pathways. Altogether, these studies indicate the important role of claudins contributing to drug resistance in cancer cells. The pictorial representation of the role of claudins as a stem cell regulator and its impact in chemoresistance is shown in Figure 2.

## 5. Claudins in Prognosis

Emerging data defining mechanisms through which claudins augment cancer metastasis provides the rationale for exploring claudins as prognostic factors and therapeutic targets in cancer. The importance of claudins is established using cancer cell models, mouse models, and human patient samples. Target molecules for cancer surveillance in high-risk populations are desperately warranted. As a vital emerging modulator in molecular or cellular pathways related to cancers, claudins could be targeted or used as biomarkers for prognosis, diagnosis, and treatments. A number of recent studies have projected a role for claudins as key prognosis factors in cancers. In one of the study Lechpammer et al. [104] demonstrated the potential of claudins as a diagnostic and prognostic factor in renal cell carcinoma. Claudin-1, -3, -4, -7, and -8 were studied in human renal cell carcinomas and oncocytomas. The data from their research showed an inverse correlation between claudin-3 and -4 expression with overall survival in clear cell renal cell carcinomas, and these claudins could be considered for prognosis in renal cell carcinomas. Claudin-7 and 8 can be implied as useful markers in the identification of renal cell carcinomas from oncocytomas [105]. 

Claudin-6 was reported as a prognosis factor in NSCLC patients. In this report, the patients with low claudin-6 had a lower survival rate than the patients with high claudin-6. [91] reported low claudin-6 as an independent indicator of prognosis in NSCLC patients. In this study, they documented low claudin-6 in 61 of 123 NSCLC tissue samples, and patients with low claudin-6 expression correlated with lower survival rates than those with high claudin-6 expression. The influence of claudin-3, claudin-7, and claudin-18 in gastric cancer patients were also studied [106]. Claudin-3 and claudin-7 were expressed in 25.4% and 29.9% of the gastric cancer tissues, respectively. However, 51.5% of gastric cancer tissues exhibited reduced expression of claudin-18. Claudin-7 expression correlated with shorter overall survival in gastric cancer patients, while the overall survival was increased in patients with claudin-18 expression. Recently, claudin-3 and -7 are also considered as novel prognostic factors in triple-negative breast cancer (TNBC) through its aberrant immunohistochemical expressions [107]. Claudin-3 cytoplasmic expression is an indicator of poor survival in triple-negative breast cancer. In addition, epigenetic modifications of claudins are reported to be a promising prognosis marker of various cancers. Zhenzhen et al. [106] recently demonstrated that the methylation of claudin-3 is a prognostic factor in gastric adenocarcinoma.

Further, the serum levels of claudin-7 among patients with colorectal cancer (CRC) was significantly reduced and correlated with high tumor stage and high carcinoembryonic antigen levels [108]. Claudin-7 was found to be downregulated in CRC, as reported by Bhat et al. [42], and associated with diminished EMT and tumor progression. These studies give a strong rationale to consider claudin-7 as a biomarker for predicting the development, proliferation, and prognosis of CRC. A claudin-low molecular subtype of breast cancer has been described with a concomitant upregulation of several EMT markers and an enrichment in stem cell features [109]. In an interesting article by Danzinger et al., the importance of claudin-3 in triple-negative breast cancer (TNBC) was documented. It was reported that claudin-3 expression was correlated with a Breast cancer type 1 (BRCA1) mutation [107]. This could help in guiding the decision for BRCA testing for triple-negative breast cancer (TNBC). Also, the expression of claudin-11 has been suggested as a biomarker for advanced-stage cutaneous squamous carcinoma, and reflects the distinct stages of tumor development and differentiation [110]. The clinical significance of claudin-11 was addressed in Laryngeal Squamous Cell Carcinoma (LSCC) by Nissinen et al. [110]. In this study, elevated promoter methylation of claudin-11 in tumor tissues was observed. Patients with lymph node metastasis with an advanced clinical stage showed more methylation in the claudin-11 promoter, which associated with poor overall survival of LSCC patients. In TNBCs, claudin-1, -3, -4, and -7 higher expression rates are more frequent than in other subtypes [111]. Claudin-4 high/claudin-1 low, claudin-4 high/claudin-7 low, and claudin-4 high/claudin-1 low/claudin-7 low types were also significantly correlated with lymph node metastasis, and showed worse survival. Apart from this, a recent article from Upadhaya et al. documented the therapeutic potential of claudin-1 in oral epithelial dysplasia and oral squamous cell carcinoma [112]. Overall the differential expression pattern of claudins may reflect the distinct stages of tumor development and differentiation and have been implied as prognostic factors for early determination of the tumor state.

## 6. Claudins as Therapeutic Agents

So far, over 100 monoclonal antibody (mAb) products are in clinical trials [113]. In an oncology setting, these monoclonal antibodies can mediate antibody-dependent cellular cytotoxicity (ADCC) and complement-dependent cytotoxicity (CDC) against cancer cells [114]. There is a long-lasting history of antibody-mediated targeting of claudin-1 against hepatitis C virus (HCV) infections, and wherein many researchers have provided proof for the importance of claudins in HCV infections as viral entry point [115,116]. A study by Fofana et al. [117] designed monoclonal antibodies against claudin-1 to combat HCV entry. It was promising to see the antibodies raised against claudin-1 was able to block HCV entry. A recent study by Colpitts et al. has documented the humanization of a claudin-1-specific monoclonal antibody and was investigated in a large panel of primary human hepatocytes, and was found to be very promising for clinical HCV prevention and cure [118]. These studies hold significance because these antibodies could prevent HCV infection after liver transplantation, and virus spread in chronically infected patients. These antibodies are now being tested in cancer models. Claudins, as a potential target in antibody-based therapies for carcinomas, was investigated by Offner et al. [119]. In this study, the antibodies were raised against the extracellular domains of claudin-1, -3, and -4. Recently Romani et al. engineered a fully human anti-claudin-3 IgG1 antibody (IgGH6) [120], which is specific to claudin-3 and no cross-reactivity with other claudins was observed. Recent work by Cherradi et al. [121] investigated the importance of claudin-1 in different colorectal cancer (CRC) molecular subtypes. There is a differential expression pattern of claudin-1 based on the subtype. A murine monoclonal antibody against the extracellular part of human claudin-1 (6 F6 mAb) was designed and generated, which was specifically able to pick claudin-1 positive CRC cell lines, and no other cross-reactivity was observed. Furthermore, 6 F6 mAb was able to combat colony formation, xenograft growth and metastasis of claudin-1 positive CRC cells suggesting its utility as a therapeutic. Fujiwara et al. recently targeted claudin-4 in CRC using an anti-claudin-4 extracellular domain antibody [122]. The efficacy of the anti-claudin-4 antibody is promising and observed to enhance the anti-tumorigenic potential of 5-fluorouracil (FU) and anti-EGFR antibodies. These works demonstrate the proof of concept for exploiting claudins as targets for monoclonal antibodies in therapies.

Some of the monoclonal antibodies against claudins, including anti-claudin-18.2 (IMAB362-claudin-18.2) and the anti-claudin-6 (IMAB027-claudin-6), have also found their way into clinical trials [123]. Claudin-18.2 is expressed on the outer cell membrane of gastric cancer cells and binds to monoclonal antibodies. The IMAB362 was proven to be clinically safe as the patients were devoid of any side effects. Also, IMAB027 is in an ongoing clinical trial for recurrent advanced ovarian cancer (NCT02054351), and patients have not demonstrated any adverse effects [123]. Clinical trials for claudiximab (claudin-18 targeting) in advanced gastroesophageal cancer patients are also underway [124]. Recently, claudiximab was reported to be a first-in-class chimeric monoclonal antibody for the treatment of gastric cancer targeting claudin-18, which is an important factor in gastric cancer metastasis. This is just the beginning of an exciting journey and more research is warranted to revolutionize claudins targeted monoclonal antibodies in cancer therapy.

Another avenue to exploit Claudins as a therapeutics is their ability to behave as receptors for microbes. *Clostridium perfringens* enterotoxin (CPE) has the potential to bind with claudin receptors. CPE binds to the C-terminal CPE domain at both the first and second extracellular loops (ECL-1 and ECL-2) of claudins [125]. The affinity of CPE to claudins causes a pore leading to calcium influx responsible for host cell death. The claudin–CPE interaction is gaining significance in receptor decoy therapeutics for potential applications in gastrointestinal disease, cancer therapy/diagnoses, and drug delivery [125]. Claudin-3 and claudin-4 have been widely demonstrated to function as CPE receptors [126,127]. The binding ability of CPE to claudins, especially claudin-3 and claudin-4, has raised a great opportunity to target cancers with dysregulated claudin-3 and -4 cancers, especially breast, ovarian, and pancreatic cancers. The binding of CPE to claudin-3 and -4 was documented to induce dose-dependent cytolysis in breast cancer cells expressing claudin-3 and -4 [128]. Recent studies have exploited the CPE mediated targeting of claudin-3 and 4 cancers to target therapy-resistant ovarian cancer, pancreatic, and breast cancer xenografts possessing increased expressions of claudin-3 and -4. In one of the studies, the possibility of CPE binding claudin-3 as a visualization tool for identifying of micrometastatic chemotherapy-resistant ovarian cancer has been demonstrated [129]. The applicability of CPE, claudin-3, and -4 interactions is exploited in gene therapy against colon cancer. Recombinant (recCPE) and optimized CPE expressing vector (optCPE) were demonstrated to have a cytotoxic effect in claudin overexpressing colon cancer cells [130,131]. Further, the recent identification of the crystal structure of claudin-9 revealed that human claudin-9 has high-affinity for the CPE receptor and treatment with CPE caused cell death in human claudin-9 expressing cells [132]. In continuation of these studies, an interesting approach of nanoparticle-based targeting of cancer cells was documented by researchers, wherein the C-terminus of the CPE was conjugated to gold nanoparticles (AuNPs). This combination binds to claudin expressing tumor cells and kills the cells using gold nanoparticle-mediated laser perforation (GNOME-LP) technique [133,134]. Thus, the clinical relevance and functional importance of claudins in diverse cancers make them potential therapeutic targets.

## 7. Claudins as a Visualization Tool

The use of monoclonal antibodies against claudins have also been utilized in imaging modalities. Recently, claudin-4 was studied as an imaging tool for x-ray computed tomography (CT) in the prognosis of pancreatic ductal adenocarcinoma (PDAC) [135]. Claudin-4 is a known biomarker in PDAC detection. In this study, researchers reported a novel radiolabeled anti-claudin-4 monoclonal antibody in detecting PDAC using single-photon emission computed tomography (SPECT) imaging. The results showed promising uptake of anti-claudin-4 monoclonal antibody by PDAC tumors and were helpful in early detection and characterization of PDAC malignancy. Also, the researcher later targeted the extracellular domain of claudin-4 (4D3) with monoclonal antibodies (4D3) in combating bladder and lung cancer [136]. 

**Figure 3:**
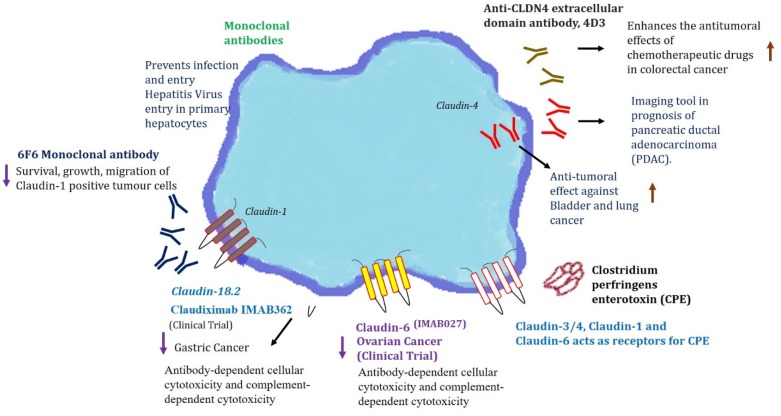
Claudins as an employable platform for prognostic, diagnostic, and therapeutic targets. The upward arrow indicated upregulation and downwards arrow indicated downregulation of the mentioned signaling events.

Colonoscopic aided screening and polyp and tumor removal have led to the reduced incidence and mortality of colorectal cancer (CRC). However, the lack of specificity is a major pitfall in these approaches and makes them less effective. It is especially difficult to detect the regions of flat dysplasia or serrated polyps, which also possess malignant potential. Thus, a targeted approach for advanced endoscopic techniques is a cornerstone requirement. A promising approach was recently demonstrated for the real-time endoscopic imaging of colonic adenomas [137]. In this study, the researchers exploited claudin-1 as a potential target in endoscopic imaging of colonic adenomas. As claudin-1 is highly expressed in the early development of CRC, endoscopic imaging might be useful for detecting either polypoid or flat precancerous lesions that are difficult to visualize [138]. Peptide (peptide sequence—RTSPSSR), specific to claudin-1, was developed against the extracellular loop of claudin-1. This peptide showed greater intensity for human adenomas, hyperplastic polyps and sessile serrated adenomas thus proposing the possibility of using claudin-1 peptide aided endoscopic imaging for the future clinical translation to detect precancerous lesions. Recently another study by our group demonstrated the significance of claudin-1 as a useful target for near-infrared antibody-based imaging for visualization of colorectal tumors [138]. When animals injected with colon cancer cells subcutaneously were imaged using claudin-1 antibody conjugated LI-COR IR800DyeCW through a LI-COR Pearl Trilogy Fluorescence Imaging System, the system was able to target tumors specifically. These studies pave the way for using claudins as a tool for fluorescence-guided surgery, which will help in more specific targeting of the tumors in a stage-specific manner. A comprehensive representation encompassing the role of claudins and the monoclonal antibodies against claudins as therapeutic and detection tools is given in Figure 3, and the role of claudins as a therapeutic, prognostic, and detection agents is tabulated in Table 3.

## 8. Future Perspectives

The quest for prognostic, diagnostic, and therapeutic markers for many cancers is of high importance. More reliable and earlier detection markers have implications for diagnostic and therapeutic targeting. As the role of claudins in the regulation and enrichment of cancer stem cells and chemo-resistance becomes obvious, targeting claudins for diminishing cancer stem cells, which are cancer-propagating subsets of malignant cells, would be very useful. The potential of the claudin–cancer stem cell axis provides great potential for combating invasive, metastatic, and drug resistance phenotypes of various cancers. Future studies focusing on the role of claudins in cancer stem cells will be warranted to specifically target these populations to curb down residual tumor cells left after standard therapies.

Claudins are gaining their importance as detection and therapeutic agents. Future engineering of more monoclonal antibodies against claudins will have potential applications in targeted therapy, and claudin assisted endoscopy, imaging of various tumors. Also, the antibody-based detections will provide ample opportunity for the early diagnosis of any inflammatory diseases before they reach cancer status. The ongoing clinical trials for monoclonal antibodies against claudins might lead to claudin directed immunotherapies. Recently, small molecules inhibitors have been gaining more attention in cancer biology, as they aid in targeted therapy. No known small molecule inhibitors are currently being researched for claudins. Thus, in the future, screening for more potent inhibitors against claudins is warranted. Overall, to strengthen the therapeutic window of claudins, a more translational view of claudins by researchers is warranted.

## Figures and Tables

**Table 1:** Claudins as tumour promotor/suppressor.

Claudins Subtype	Cancer Type	Proto-Oncogene	Reference
Claudin-6	Gastric cancer	Tumour promotor	[25]
Claudin-1	Colon cancer	Tumour promotor	[40,45]
Claudin-3	Ovarian cancer	Tumour promotor	[22]
Claudin-4	Ovarian cancer	Tumour promotor	[22]
Claudin-6	Breast cancer, Gastric cancer	Tumour promotor	[26,27]
Claudin-7	Colon cancer	Tumour promotor	[46]
Claudin-2	Lung cancer	Tumour promotor	[28]
Claudin-1	Gastric cancer	Tumour suppressor	[47]
Claudin-1	Lung cancer	Tumour suppressor	[29]
Claudin-3	Ovarian cancer	Tumour suppressor	[31]
Claudin-4	Ovarian cancer	Tumour suppressor	[31]
Claudin-7	Lung cancer	Tumour suppressor	[30]
Claudin-11	Gastric cancer	Tumour suppressor	[48]
Claudin-2	Osteosarcoma	Tumour suppressor	[49]

**Table 2:** Claudins and stemness.

Claudin Subtype	Stem Cell Related Functions	References
Claudin-6	Early marker in embryonic stem cell.High expression in undifferentiated human pluripotent stem cells (hPSCs). Trigger epithelial morphogenesis in mouse stem cells.	[73,74]
Claudin-1 and 2	Known to regulate the β-Catenin-TCF/LEF signaling pathway to regulate CSC.	[81]
Claudin low subtype in breast cancer	Enriched in stem cells and more EMT.	[83]
Claudin-3	Regulation on cancer stemness and chemoresistance in non-small cell lung cancer (NSCLC).	[84]
Claudin-18	Triggers lung enlargement and parenchymal expansion by restrictions on stem/progenitor cell proliferation.	[85]
Claudin-2	Enrich ALDH^High^ cancer stem-like cells in heterogeneous colorectal cancer cell populations.	[86]

**Table 3:** Claudins as prognostic, therapeutic and detection agents.

Claudins Subtype	Disease Type	Therapeutic Agent	Clinical Application	Reference
Claudin-1	Hepatitis C virus infection	Residues within the first extracellular loop.	Hepatitis C virus co-receptor.	[139]
		Humanization of a claudin-1-specific monoclonal antibody.	Clinical prevention and cure of Hepatitis C virus(HCV) infection.	[118]
Claudin-6	Ovarian cancer	*Clostridium perfringens* enterotoxin (CPE) cytotoxicity.	CPE-mediated cytotoxicity in Ovarian cancer.	[127]
Claudin-3	Ovarian cancer uterine carcinomas	Human anti-claudin-3 IgG1 antibody.	Candidate for antibody-drug conjugate therapeutic applications.	[120,140]
Claudin-1	Colon cancer	Human claudin-1 (6F6 mAb).	Suppressed survival, growth, and migration of claudin-1 positive cells. Suppressed tumor growth and liver metastasis formation.	[121]
Claudin-4	Colorectal cancer	Anti-claudin-4 extracellular domain antibody.	Enhancer of anti-tumoral effects of chemotherapeutic agents.	[122]
Claudin-4	Pancreatic Cancer (PDAC)	Indium-111 tagged anti-claudin-4 monoclonal antibody.	X-ray computed tomography sided detection of PDAC.	[135]
Claudin-18.2	Gastric and gastroesophageal junction cancer	Chimeric monoclonal antibody that binds to claudin-18.2 (NCT03504397)	Cell death through antibody-dependent cellular cytotoxicity and complement-dependent cytotoxicity.	[123]
Claudin-4	Pancreatic cancer	Claudin-4 binder C-CPE 194	Enhances Tazeffects of anticancer agents via a MAPK pathway.	[141]
Claudin-3 and 4	Prostate cancer	Claudin-3 and claudin-4 targeted *Clostridium perfringens* protoxin	Selectively cytotoxic to PSA-producing prostate cancer cells.	[126]
Claudin-1	Colon cancer	Peptide RTSPSSR, specific to claudin-1 against the extracellular loop of claudin-1.	Specific to human adenomas, hyperplastic polyps, and sessile serrated adenomas.	[137]
Claudin-1	Colon cancer	Claudin-1 antibody conjugated with LI-COR IR800DyeCW	Near-infrared antibody-based imaging for visualization of colorectal tumors.	[138]
Claudin-9	Hepatitis C virus infection	Residues N38 and V45 in the first extracellular loop (EL1) of claudin-9 are responsible for HCV entry. Also found in PBMS (peripheral blood mononuclear cell) contributing to extrahepatic HCV infection.	It can be implicated in the development of drugs to block HCV entry into the liver and peripheral blood mononuclear cell (PBMS).	[142]
Claudin-11	Gastric Cancer	Hyper-methylation of claudin-11 promotor region leads to significant downregulation in gastric cancer.	Identification of the associated signaling cascades might lead to novel approaches in diagnosis and therapy for gastric cancer.	[48]
Claudin-7	Non-small cell lung cancer (NSCLC)	Reduced expression—Poor outcome Claudin-7 low NSCLC—Poor survival. Claudin-7 high NSCLC—High Survival.	Biomarker and a potential therapeutic target in patients with NSCLC.	[143]
Claudin-7	Epithelial Ovarian cancer	Claudin-7 transcripts were significantly enhanced in epithelial ovarian carcinoma patients. Silencing claudin-7 displayed enhanced sensitivity to Cisplatin treatment.	Independent prognostic factor and a key protein in regulating response to platinum-based chemotherapy in the treatment of epithelial ovarian cancer (EOC).	[144]
Claudin-2	Irritable bowel disease (IBD)	Anti-claudin-2 mAb 1A2	Prevent *cis*- and *trans*-interactions of claudin-2, attenuating the formation of leaky tight junction (TJ) seals.	[145]
